# Predictors and moderators of response to enhanced cognitive behaviour therapy and interpersonal psychotherapy for the treatment of eating disorders

**DOI:** 10.1016/j.brat.2016.07.002

**Published:** 2016-09

**Authors:** Zafra Cooper, Elizabeth Allen, Suzanne Bailey-Straebler, Shawnee Basden, Rebecca Murphy, Marianne E. O’Connor, Christopher G. Fairburn

**Affiliations:** a Oxford University, Department of Psychiatry, UK; b London School of Hygiene and Tropical Medicine, Department of Medical Statistics, UK

**Keywords:** Eating disorders, Predictors, Moderators, Outcome, Enhanced cognitive behavioural therapy, Interpersonal psychotherapy

## Abstract

Consistent predictors, and more especially moderators, of response to psychological treatments for eating disorders have not been identified. The present exploratory study examined predictors and moderators of outcome in adult patients who took part in a randomised clinical trial comparing two leading treatments for these disorders, enhanced cognitive behavioural therapy (CBT-E) and interpersonal psychotherapy (IPT). Four potentially important findings emerged. Firstly, patients with a longer duration of disorder were less likely to benefit from either treatment. Second, across the two treatments the presence, at baseline, of higher levels of over-evaluation of the importance of shape predicted a less good treatment outcome. Third DSM-IV diagnosis did not predict treatment outcome. Fourth, with the exception of patients with baseline low self-esteem who achieved a better outcome with CBT-E, it was generally not possible to identify a subgroup of patients who would differentially benefit from one or other treatment.

## Highlights

• Investigated predictors and moderators of outcome in CBT-E and IPT for eating disorders.
• Those with longer duration of disorder less likely to benefit from either treatment.
• In both treatments high levels of shape concern associated with less good outcome.
• DSM-IV diagnosis did not predict treatment outcome.
• Generally, not possible to identify those who differentially benefit from CBT-E or IPT.

## 1 Introduction

Knowledge of the predictors of response to treatments and the factors that moderate the effects of treatment on outcome have the potential to lead to more precise clinical decision-making and to promote personalised medicine (Kraemer, 2013).

While considerable progress has been made in the development and evaluation of psychological treatments for eating disorders, less is known about predictors and moderators of treatment response. Consistent predictors, and more especially moderators, of response have not been identified (Agras et al., 2014, Agras et al., 2000, Grilo et al., 2012, Iacovino et al., 2012, Le Grange et al., 2014, Le Grange et al., 2012;; Le Grange et al., 2008, Steinhausen and Weber, 2009, Thompson-Brenner et al., 2013, Vall and Wade, 2015).

In the present study we examined predictors and moderators of treatment outcome in patients who took part in a randomised controlled trial (RCT) comparing enhanced cognitive behaviour therapy (CBT-E) and interpersonal psychotherapy (IPT) in the treatment of adult patients with an eating disorder. Patients with any eating disorder diagnosis were eligible provided that their body mass index (BMI) was over 17.5 and below 40.0. There have been no previous studies with this design. The results of this RCT indicated that at the end of treatment the remission rate was higher among those who had received CBT-E than IPT (CBT 65.5%; IPT 33.3%). At 60-week post-treatment follow-up the IPT remission rate had increased to 49%, but there was still a significant advantage for CBT-E with a remission rate of 69.4% (Fairburn et al., 2015). Nevertheless, the study demonstrated that IPT is also a viable treatment for this large group of patients with an eating disorder.

In designing the present study we decided to focus on the predictors and moderators of outcome at 60 weeks post-treatment rather than on those immediately following treatment. This decision was made because it was only by this point that the effects of IPT had been fully expressed (Fairburn et al., 2015), a finding that is consistent with those from two earlier studies of IPT (Agras et al., 2000, Fairburn et al., 1993, Fairburn et al., 2015) Were we to have focused on the patients’ state immediately after treatment, this would have biased the findings against IPT and might have generated misleading results in terms of predicting true clinical benefit.

The present study followed the recommendation of Kraemer and colleagues (Kraemer, Wilson, Fairburn, & Agras, 2002) in making use of an RCT to explore moderators of treatment outcome.

## 2 Method

### 2.1 Design

Predictors and moderators of treatment outcome were sought using data from a RCT comparing CBT-E and IPT in the treatment of adult patients with an eating disorder (Fairburn et al., 2015). Eligible patients were randomised to either CBT-E or IPT delivered over 20 weeks. They were then entered into a closed follow-up period lasting 60 weeks, during which they received no additional treatment unless it was judged essential on clinical grounds. The predictor and moderator analyses focused on the patients’ clinical state at 60-week follow-up.

### 2.2 Sample

Consecutive patients referred to a catchment area eating disorder clinic were eligible to take part if: they had a clinically significant eating disorder; were aged 18–65 years; had a BMI over 17.5 and under 40.0; and they provided written informed consent. Exclusion criteria were: receipt of prior treatment closely resembling CBT-E or IPT; a co-existing general psychiatric disorder that precluded eating disorder-focused treatment; medical instability or pregnancy; and not being available for treatment.

One hundred and thirty eligible participants were randomised, 65 to CBT-E and 65 to IPT. Their diagnoses were as follows: BN – 53 participants (40.8%); BED – 8 participants (6.2%); and “other eating disorder” (OED) – 69 participants (53.1%). Details of the sample and the CONSORT diagram are provided in the main outcome paper (Fairburn et al., 2015).

### 2.3 Treatments

Both treatments were delivered in 20 50-min sessions, preceded by one 90-min preparatory session, and followed by a review session 20 weeks after the completion of treatment.

#### 2.3.1 Enhanced cognitive behaviour therapy

CBT-E is designed to address eating disorder psychopathology whatever the eating disorder diagnosis. It is personalised to match the eating disorder psychopathology of the individual patient (Fairburn, 2008). The default (“focused”) form of the treatment was used, concentrating on the modification of eating habits, weight control behaviour and concerns about eating, shape and weight.

#### 2.3.2 Interpersonal psychotherapy

IPT is a short-term psychological treatment designed to identify and address current interpersonal problems (Klerman, Weissman, Rounsaville, & Chevron, 1984). Although originally a treatment for depression, it has been adapted to make it suitable for the treatment of BN (Fairburn, 1993) and BED (Wilfley et al., 2002, Wilson et al., 2010) and has been shown to be an effective treatment for both of these conditions. The form of IPT used in the present study has been described in detail elsewhere (Murphy, Straebler, Basden, Cooper, & Fairburn, 2012).

#### 2.3.3 Therapists

Treatment was provided by three therapists, two clinical psychologists and a psychiatric nurse practitioner. All three had generic clinical experience as well as experience treating eating disorders. They received six months’ training in both treatments from ZC and CGF prior to the start of the study and subsequent weekly supervision (also from ZC and CGF) including the auditing of supervisor-selected audio recordings. Further details concerning treatment fidelity and its auditing are provided in the main outcome paper.

### 2.4 Assessment

#### 2.4.1 Eating disorder features

These were assessed using the 16th edition of the Eating Disorder Examination interview (EDE) (Fairburn, Cooper, & O’Connor, 2008). Operational DSM-IV diagnoses of BN and BED (American Psychiatric Association, 1994) were generated from the EDE ratings, with the remaining participants being given the diagnosis of OED. The primary (continuous) outcome used in this study was the severity of eating disorder features as measured by the EDE global score.

#### 2.4.2 General psychiatric features

The Structured Clinical Interview for DSM-IV (First, Gibbon, & Williams, 1997) was used at baseline to identify the presence of co-existing general psychiatric disorders, and the level of depressive features was measured using the Beck Depression Inventory (BDI) (Beck, Ward, Mendelson, Mock, & Erbaugh, 1961).

#### 2.4.3 Weight and body mass index

Weight and height were measured at baseline. Weight was measured using a beam balance scale and height was measured using a wall-mounted stadiometer. BMI was calculated using these measurements.

#### 2.4.4 Self-esteem

The Rosenberg Self-esteem scale (RSE; (Rosenberg, 1979)) was used to measure self-esteem at baseline.

#### 2.4.5 Social functioning

The self-report version of the Social Adjustment Scale (SAS) (Weissman & Bothwell, 1976) was used at baseline to assess social role performance in several domains.

### 2.5 Data analysis

As recommended for moderator analyses (Wallace, Frank, & Kraemer, 2013), we restricted the analyses to a primary outcome used in the RCT. In order to maximise our power to detect moderator effects we chose the continuous outcome variable, the severity of eating disorder features, as measured by the EDE global score. Mixed effect logistic regression models were fitted with a random effect at the individual level. Data from all time points were included in all analyses so that the data from all participants contributed to the analysis, even if there were missing data at follow up. The models had the potential moderator, treatment, time, and treatment × moderator interaction (at each time point) and the baseline measure of the outcome as independent variables. Moderation was considered to have occurred when a significant moderator × treatment interaction was found indicating that the treatment worked differently for various levels of the moderator. All analyses were performed in Stata.

The variables examined, first as potential predictors and then as moderators of outcome, were age, baseline eating disorder characteristics (baseline BMI, lowest adult BMI, duration of disorder, history of anorexia nervosa, DSM-IV diagnosis, binge eating, self-induced vomiting, importance of shape (EDE item score), importance of weight (EDE item score)); associated psychopathology (presence of any Axis I psychiatric disorder, depression (BDI), taking psychotropic (antidepressant) medication); self-esteem (RSE) and social functioning (SAS). Continuous variables were categorised to enhance their clinical relevance and to avoid the assumption that predictors and moderators operate in a linear fashion (Kraemer, 2016).

Despite the large number of tests conducted, a significance cut off of around p < 0.05 was used as a guide in interpreting our findings. This is consistent with our aim of generating, rather than testing hypotheses.

## 3 Results

### 3.1 Predictors of outcome

Two baseline variables were associated with the severity of eating disorder features at 60 week follow up (see Table 1). These were: longer duration of eating disorder and higher levels of importance of shape. Those whose eating disorder duration at baseline was equal to or greater than 8 years had more severe eating disorder features at 60 week follow up than those who had a disorder of shorter duration (0.51, 95% Cl 0.03, 0.59, p = 0.04). Baseline levels of importance of shape equal to 5 or 6, in contrast with those below 5, were also associated with a greater severity of eating disorder features at 60 weeks (0.64, 95% Cl 0.11,1.16, p = 0.02).

### 3.2 Moderators of outcome

The effect of CBT-E as compared to IPT on outcome was different depending on baseline level of self-esteem. As can be seen from Table 2 at 60 week follow up those with low self-esteem (RSE <13) at baseline who received CBT-E had lower scores on the severity of eating disorder features (EDE global score) than those who received IPT (−0.70, 95% Cl −1.31, −0.08). For those whose baseline self esteem scores were higher (≥13) there was no relative advantage for either treatment (0.09, 95% Cl −0.47, 0.66). There were no other baseline characteristics that identified a subgroup of patients who differentially responded to either treatment (see Table 2).

## 4 Discussion

This hypothesis-generating study explored predictors and moderators of treatment response to CBT-E and IPT in a sample of adult patients with an eating disorder. The sample included patients with BN, BED and OED provided that their BMI was over 17.5 and below 40.0. The analyses were restricted to a primary continuous outcome variable used in the RCT. The use of a single outcome variable followed recommendations for moderator analyses (Wallace et al., 2013) and the choice of a continuous variable was made to maximise the power of the analyses. The outcome variable was ‘severity of eating disorder features’ defined as global EDE score. The analyses focused on outcome at 60-week follow up as the effects of IPT take up to 12 months after treatment to be fully expressed. The follow-up period was closed and as a result there was little contamination from exposure to further treatment (Fairburn et al., 2015).

Duration of disorder and over-evaluation of the importance of shape predicted response across the two treatments. Patients were more likely to have eating disorder features if, at baseline, they had a longer duration of disorder and high levels of over-evaluation of shape.

Certain of these findings are consistent with those from other studies. For example, lower baseline levels of over-evaluation of the importance of shape or weight have been reported to predict remission at the end of treatment in adults with BED (Grilo et al., 2012) and in adolescents with BN (Le Grange et al., 2008). Over-evaluation of the importance of shape and weight has also been associated with the persistence of symptoms over 5 years in a naturalistic study of BN (Fairburn et al., 2003) and to predict relapse after remission in treated samples with BN (Fairburn et al., 1993, Keel et al., 2005). Also, in general, a longer duration of disorder and an earlier age of onset have been associated with less good treatment outcomes at end of treatment and at follow-up in patients with BN and BED, although the effect sizes are small (Vall & Wade, 2015).

Only one moderator of treatment was found, indicating that for those patients with low self-esteem CBT-E rather than IPT was associated with a better outcome at 60 week follow up whereas for those with higher levels of self-esteem there was no relative advantage for either treatment. There were no other baseline characteristics that identified a subgroup of patients who would differentially benefit from one or other treatment. The study therefore provides little guidance for matching patients to these two treatments.

Four potentially important findings emerge from the study. Firstly, those with a longer duration of disorder at baseline, specifically 8 or more years, were less likely to benefit from treatment. At 60 week follow up they had higher levels of eating disorder features as compared to those with a shorter duration of disorder at the start of treatment. Early intervention and treatment is therefore likely to be beneficial. Second, the presence, at baseline of the highest levels of over-evaluation of the importance of shape predicted a less good treatment outcome. This may suggest a need to improve strategies for addressing this form of psychopathology or perhaps point to some further features of these patients that require investigation because of their association with a less good outcome. Third, DSM- IV diagnosis did not predict treatment outcome. Irrespective of whether patients received CBT-E or IPT, eating disorder severity at 60 weeks was not determined by their diagnostic status at baseline. This finding is consistent with a transdiagnostic perspective on the classification and treatment of the eating disorders. Fourth, with one exception it was not possible to identify patients who would differentially benefit from either treatment and therefore little guidance could be provided for matching specific subgroups of these patients to the treatments.

## Conflict of interest

None.

## Figures and Tables

**Table 1:** Predictors of outcome (Global EDE score) at 60 week follow up.

Predictor	60 week follow up	
Mean difference (95% Cl)	P
Age ≥ 24	0.27 (−0.21,0.75)	0.27
Diagnosis (other ED/BN)	−0.23 (−0.72, 0.26)	0.37
History of AN	0.39 (−0.15, 0.93)	0.16
Presence of binge eating – OBE ≥4 (1 month)	−0.00 (−0.63, 0.63)	0.99
Presence of self induced vomiting ≥4 episodes (1month)	−0.16 (−0.65, 0.34)	0.53
Duration of disorder ≥ 8 years	0.51 (0.03, 0.59)	0.04
Lowest BMI ≥ 19	0.02 (−0.46, 0.51)	0.93
Baseline BMI ≥ 21.7	0.29 (−0.19, 0.77)	0.24
Importance of weight ≥ 5	0.32 (−0.18, 0.82)	0.21
Importance of shape ≥ 5	0.64 (0.11, 1.16)	0.02
Presence of any co-morbid axis 1 disorder	0.25 (−0.25, 0.75)	0.33
Taking anti-depressant medication	0.07 (−0.42, 5.6)	0.78
BDI ≥ 20	0.30 (−0.17, 0.78)	0.21
SAS (social functioning) ≥ 1.47	0.32 (−0.17, 0.81)	0.20
RSE (self-esteem) ≥ 13	−0.07 (−0.56, 0.43)	0.80

**Table 2:** Moderators of outcome at 60 week follow up.

60 week follow up			
Moderator- categorical exposures		Mean difference (95% Cl)	p
Age	<24	−0.50 (−1.06, 0.06)	0.18
≥24	−0.03 (−0.61, 0.56)	
Diagnosis	Bulimia nervosa	−0.52 (−1.15, 0.11)	0.27
Other Eating disorder (including BED)	−0.12 (−0.65, 0.42)	
History of AN	yes	−0.54 (−1.06, −0.01)	0.07
no	0.15 (−0.54, 0.84)	
Presence of binge eating	OBE ≥ 4 (1 month)	−0.11 (−1.06, 0.83)	0.68
OBE < 4 (1 month)	−0.32 (−0.81, 0.17)	
Presence of self induced vomiting	SIV ≥ 4 (1 month)	−0.22 (−0.87, 0.42)	0.81
SIV < 4 (1 month)	−0.31 (−0.84, 0.22)	
Duration of disorder	<8	−0.23 (−0.79, 0.33)	0.87
≥8	−0.29 (−0.88, 0.31)	
Lowest BMI	<19	0.01 (−0.54, 0.56)	0.09
≥19	−0.59 (−1.19, 0.02)	
Baseline BMI	<21.7	−0.08 (−0.65, 0.49)	0.27
≥21.7	−0.47 (−1.06, 0.12)	
Importance of weight	<5	−0.27 (−0.98, 0.44)	0.77
≥5	−0.38 (−0.91, 0.14)	
Importance of shape	<5	0.00 (−0.77, 0.77)	0.27
≥5	−0.45 (−0.95, 0.04)	
Presence of any co-morbid axis 1 disorder	yes	−0.28 (−0.82, 0.25)	0.98
no	−0.27 (−0.94, 0.39)	
Anti-depressant medication	yes	−0.25 (−0.82, 0.33)	0.75
no	−0.36 (−0.95, 0.23)	
Presence of any co-morbid axis 1 disorder	yes	−0.28 (−0.82, 0.25)	0.98
no	−0.27 (−0.94, 0.39)	
BDI	<20	−0.11 (−0.69, 0.48)	0.35
≥20	−0.44 (−1.01, 0.13)	
SAS	<1.47	−0.40 (−0.98, 0.19)	0.36
≥1.47	−0.07 (−0.67, 0.53)	
RSE	<13	−0.70 (−1.31, −0.08)	0.03
≥13	0.09 (−0.47, 0.66)	
